# Awareness and monitoring of infection control practices among healthcare workers in three primary health centers in India

**DOI:** 10.3205/dgkh000514

**Published:** 2024-11-05

**Authors:** Manjushree V. Mulay, Shraddha D. Naik, Anupama S. Wyawahare, Swati M. Mahajan, Smita S. Kulkarni

**Affiliations:** 1 Department of Microbiology, MGM Medical College and Hospital, Aurangabad, Maharashtra, India

**Keywords:** infection control practices, awarness, primary health centers, healthcare workers

## Abstract

**Objective:**

This study aimed to assess the awareness of and adherence to infection control practices among healthcare workers (HCWs) in three primary healthcare centers (PHCs) near Aurangabad City, Maharashtra, India.

**Method:**

A prospective observational study over six months involved 64 HCWs from three PHCs (A, B, and C). Questionnaires and observation checklists based on guidelines from the WHO and the Systems for Improved Access to Pharmaceuticals and Services (SIAPS) were used to evaluate infection control practices across nine modules. These modules encompass health facility information, employee health, cleaning practices, hand hygiene, waste management, isolation and standard precautions, childbirth/obstetrics, sterilization, and preparation/administration of parenteral medications.

**Results:**

The study revealed varying levels of adherence to infection control practices among the three PHCs. PHC-A demonstrated strong practices with an overall score of 66%, while PHC-B and PHC-C exhibited weaker practices with 40% and 38%, respectively. Hand hygiene practices showed higher compliance at PHC-A (78%), contrasting with lower compliance observed at PHC-B (39%) and PHC-C (33%). The study also noted deficiencies in hand hygiene facilities and inconsistencies in injection administration and waste disposal practices.

**Conclusion:**

This study underscores the importance of ongoing training and targeted interventions to enhance infection control practices among HCWs in PHCs. The findings provide valuable insights for policymakers and administrators seeking to improve infection prevention measures in primary healthcare settings, contributing to better healthcare outcomes and enhanced patient safety.

## Zusammenfassung

**Zielsetzung:**

Es sollten das Bewusstsein und die Einhaltung von Infektionskontrollpraktiken bei Beschäftigten im Gesundheitswesen in drei lokalen Gesundheitszentren (PHCs) in der Nähe der Stadt Aurangabad, Maharashtra, Indien, bewertet werden.

**Methode:**

An der prospektiven Beobachtungsstudie über sechs Monate nahmen 64 Angestellte des Gesundheitswesens aus drei PHC teil. Anhand von Fragebögen und Beobachtungschecklisten, die auf den Richtlinien der WHO und dem System for Improved Access to Pharmaceuticals and Service (SIAPS) basieren, wurden die Praktiken der Infektionskontrolle in folgenden neun Modulen bewertet: Informationen über die Gesundheitseinrichtung, Gesundheit der Mitarbeiter, Reinigungspraktiken, Händehygiene, Abfallmanagement, Basishygiene und Isolierung, Entbindung, Sterilisation und Vorbereitung/Verabreichung von Parenteralia.

**Ergebnisse:**

Die Studie ergab, dass sich die drei PHCs in unterschiedlichem Maß an Infektionskontrollpraktiken hielten. PHC-A wies mit einem Gesamtergebnis von 66% eine vergleichsweise gute Praxis auf, während PHC-B und PHC-C mit 40% bzw. 38% eine schwächere Praxis aufwiesen. Die Händehygiene wurde in PHC-A (78%) besser eingehalten, in PHC-B (39%) und PHC-C (33%) dagegen weniger. Außerdem wurden Mängel bei den Möglichkeiten zur Wahrnehmung der Händehygiene sowie Unstimmigkeiten bei der Verabreichung von Injektionen und bei der Abfallentsorgung festgestellt.

**Schlussfolgerung:**

Die Studie unterstreicht die Bedeutung kontinuierlicher Schulungen und gezielter Maßnahmen zur Verbesserung der Infektionskontrollpraktiken des Gesundheitspersonals in Gesundheitszentren. Die Ergebnisse liefern wertvolle Erkenntnisse für politische Entscheidungsträger und Verwaltungsmitarbeiter, die Maßnahmen zur Infektionsprävention in der primären Gesundheitsversorgung verbessern wollen, um zu besseren Ergebnissen in der Gesundheitsversorgung und zu mehr Patientensicherheit beizutragen.

## Introduction

Healthcare-associated infections (HAIs) pose significant risks to both patients and healthcare workers (HCWs). For patients, they are a leading cause of illness and death, while for HCWs, they present occupational hazards if infection control measures are not rigorously followed during patient care. In primary health care centers (PHCs), HAIs can have serious consequences. Transmission of infections between patients and healthcare workers can also lead to outbreaks in the community [1], [2].

Preventing HAIs is a responsibility shared by all HCWs. Both medical and paramedical staff must be well versed in various measures to protect themselves. As the first point of contact for patients within the healthcare system, staff at PHCs play a crucial role. Effective prevention of HAIs in healthcare facilities hinges on successfully implementing the fundamental components of infection prevention and control programs [2], [3].

In a healthcare setting, infectious agents require three elements for transmission: a reservoir, a susceptible host, and a mode of transmission. HCWs are acutely aware that patients can be sources of HAIs. Effective prevention and control programs in healthcare organizations rely on implementing and monitoring national infection prevention measures. Therefore, healthcare professionals must be well-informed about these national infection prevention measures [2]. 

To assess infection control practices among HCWs in PHCs, we developed a questionnaire and observation checklist based on existing WHO guidelines and the infection control self-assessment tool for PHCs by SIAPS [4], [5]. This assessment evaluated compliance with infection prevention and control practices in three public healthcare facilities (A, B, C) near Aurangabad City, which offer a wide range of services.

The primary goal of this study was to ensure that all HCWs are aware of practices that can prevent the transmission of infections in PHC settings, thus contributing to the efficient functioning of the healthcare system. The rapid proliferation of multidrug-resistant organisms has escalated healthcare costs for healthcare settings and patients. Our observations, analysis, and training efforts aim to enhance infection control practices at these PHCs. This study was structured with the following objectives in mind:

- To evaluate the awareness of infection control practices among HCWs at PHCs
- To observe infection control practices at these centers based on the range of services offered by PHCs.

## Method

A prospective observational study was conducted over 6 months from Jan 1, 2020, to June 30, 2020. Approval from the institutional ethics committee was obtained before the commencement of the study. The permission to conduct the proposed study was also sought from the District Health Officer (DHO), Zilla Parishad (District Development Council), Aurangabad, India. 

The study was carried out by the Departments of Microbiology and Community Medicine at MGM Medical College and Hospital, Aurangabad, India. 64 staff members involved in patient care from three different PHCs (A, B, C) near Aurangabad City, affiliated with the Zilla Parishad, were included in the study. Visits to these three PHCs were conducted on separate days with prior permission.

During the visits, administrative and other staff were interviewed using a standardized set of questionnaires based on nine modules (Table 1 (Tab. 1)) of the infection control self-assessment tool for primary healthcare facilities [5]. Facility assessments and implementation of infection control practices were observed using a compiled checklist (Table 2 (Tab. 2)) derived from the assessment tool [5]. 

Module scoring sheet was utilized to grade each module (Table 3 (Tab. 3)). On the day of the visit, training sessions on infection control practices were conducted for all staff members using posters and charts prepared for this purpose. Statistical analysis was performed using percentages and Chi-squared tests.

## Results

28 participants were interviewed from PHC-A, 20 from PHC-B, and 16 from PHC-C, totaling 64 participants across all three PHCs. The interviews were conducted based on their respective work areas using a standardized set of questionnaires structured around nine modules, as detailed in Table 1 (Tab. 1).

Comparing the overall percentage scores across all three PHCs, PHC-A scored 66%, indicating good practices in this area. However, PHC-B and PHC-C scored 40% and 38%, respectively, indicating poor practices that require immediate attention (Table 4 (Tab. 4)).

PHC-A achieved a perfect score of 100% in module 1 (health facility information) and varied from 86% to 61% in modules 9, 4, 3, 8, 7, and 2 (preparation and administration of parenteral medications, hand hygiene, cleaning the health facility, sterilization and disinfection of equipment, childbirth/obstetrics, and employee health, respectively). Module 5 (waste management) scored 55%, while module 6 (isolation and standard precautions) scored 33% (Table 5 (Tab. 5)). 

For PHC-B and PHC-C, module 9 (preparation and administration of parenteral medications) scored 72%, and module 7 (childbirth/obstetrics) scored 53% and 48% respectively. Scores for the remaining modules were below 47% (Table 6 (Tab. 6) and Table 7 (Tab. 7)).

Hand hygiene practices were observed on five healthcare workers (1 doctor, 1 nurse, 2 auxillary nurse midwives, 1 housekeeping person) from each PHC. The observation checklist (Table 2 (Tab. 2)) included four parameters: three skill-based parameters observed during actual performance and one assessing the availability of hand hygiene materials and the condition of hand washing stations. Observations revealed varying adherence to infection control measures, with PHC-A showing the highest compliance at 66%, indicating good practices. In comparison, PHC-B and C scored 40% and 38%, respectively, indicating poorer adherence requiring attention. For correct hand washing/hand rub practices, all HCWs at PHC-A met all four criteria, while 4 healthcare workers at PHC-B and 2 at PHC-C met all criteria (Table 8 (Tab. 8)). 

Regarding hand washing facilities, each PHC was observed to have two sinks for assessing supplies and sink conditions. At PHC-A, all sinks met the criteria for supplies and sink conditions. At PHC-B, one sink met all criteria; at PHC-C, none of the sinks met the criteria for supplies and sink conditions (Table 8 (Tab. 8)). 

Regarding injection administration, HCWs across all three PHCs followed 6 of 9 criteria. Notably, none of the HCW performed hand antisepsis before injection, did not use sterile cotton or gauze to break ampoules, and did not use single-use gloves for intravenous injections (Table 8 (Tab. 8)).

For waste disposal after delivery, PHC-A adhered to all five criteria, PHC-B adhered to three criteria, and PHC C adhered to only one criterion (Table 8 (Tab. 8)). 

## Discussion

The study aimed to evaluate infection control awareness and practices among HCWs at PHCs using a structured questionnaire and observation checklist based on WHO guidelines and the Infection Control Self-Assessment Tool [5]. 

Hand hygiene adherence was highest at PHC-A (78%) compared to PHC-B (39%) and PHC-C (33%), consistent with findings from previous studies in Makkah City, Saudi Arabia [1] and Kenya [4], emphasizing the ongoing necessity for training and reinforcement of hand hygiene practices [3]. Similar conclusions were drawn by Al-Kerity et al. [2] and Al-Ahamari et al. [1], underscoring the pivotal role of hand hygiene in infection prevention among HCWs. Issues such as inadequate hand washing facilities were noted at PHC-B and PHC-C. The lack of proper facilities for hand hygiene likely contributes to the observed suboptimal compliance, highlighting the critical role of infrastructure in effective infection control. This aligns with insights from SIAPS [5] and the findings of Al-Ahamari et al. [1]. 

The evaluation of injection administration practices across the three PHCs revealed a general lack of adherence to essential requirements. HCWs frequently omitted single-use gloves for intravenous injections, failed to use sterile cotton or gauze when breaking ampoules, and inconsistently disinfected their hands before administering injections. These findings resonate with observations from primary care facilities in Abha City, Saudi Arabia [1] and the study conducted by Bedoya et al. [4], emphasizing the critical importance of proper injection practices in preventing infections.

There was variability among the three PHCs regarding waste disposal practices following a birth. PHC-A demonstrated full adherence to waste disposal criteria, whereas PHC-B and PHC-C exhibited lower compliance. Similar challenges in waste disposal practices were identified in a study conducted in Makkah City, Saudi Arabia [3]. Effective waste disposal is crucial for preventing the transmission of infections. Our study's findings align with those of Al-Ahamari et al. [1], Al-Kerity et al. [2], and Bedoya et al. [4], highlighting the need for targeted interventions, continuous training, and infrastructure improvements in infection control practices.

The assessment criteria used in our study and the study by Patwardhan et al. [6] were based on established guidelines. Our study utilized a modified tool derived from SIAPS [5], whereas Patwardhan et al. [6] employed the Government of India’s Kayakalp tool. Both studies concurred on significant gaps in healthcare facility procedures. Patwardhan et al. [6] identified deficiencies in equipment and materials for waste handlers, housekeeping, human resources, and record keeping. Both studies underscored the importance of healthcare-facility staff adhering to recommended procedures. According to Patwardhan et al., challenges included multitasking, insufficient knowledge among housekeeping staff, and neglect of hand hygiene protocols. Similarly, our study highlighted limited knowledge among housekeeping employees and waste handlers regarding cleaning supplies and methods. Our findings underscore the critical need for targeted training initiatives to address identified gaps in infection control procedures.

Conducting training sessions after each visit could effectively enhance HCWs’ understanding of and adherence to infection prevention measures. This approach has proven effective in improving infection control practices in healthcare settings, as noted by Al-Ahamari et al. [1] and Bedoya et al. [4].

Comparing our study's findings with existing literature and international standards, such as the Infection Control Self-Assessment Tool for Primary Health Care Facilities [5], provides a benchmark for evaluating the performance of the PHCs.

## Limitations

Two study limitations include potential observer bias during on-site assessments and small sample size. Future studies could consider employing more qualitative techniques and increasing the sample size to better understand the factors influencing infection control procedures.

## Conclusion

This study offers significant insights into infection control practices at PHCs, emphasizing the critical role of continuous training and interventions. Comparisons with reference studies highlight the extensive scope and challenges associated with adhering to infection control protocols.

Given the multifaceted nature of infection control, ongoing education, training, and monitoring are essential. Implementing targeted interventions based on these findings can enhance healthcare outcomes and improve patient safety within primary healthcare settings.

Policymakers and healthcare administrators seeking to enhance infection prevention practices in primary healthcare facilities may find our results valuable as a reference.

## Notes

### Authors’ ORCID

- Manjushree V. Mulay: 0000-0003-0049-7144
- Shraddha D. Naik: 0000-0001-5611-3272
- Anupama S. Wyawahare: 0000-0003-0796-6804
- Swati M. Mahajan: 0000-0002-8326-5818
- Smita S. Kulkarni: 0000-0002-8689-111X

### Author contributions

The authors Mulay and Naik have contributed equally to this work.

### Ethical approval

The research protocol titled "Awareness and Monitoring of Infection Control Practices among Healthcare Workers in Primary Health Centers" was reviewed by the Institutional Ethics Committee (IEC) of MGM Medical College and Hospital, MGM Institute of Health Sciences, Aurangabad, Maharashtra, India. After evaluation, it was determined to be suitable for research. The proposed study was approved via official communication in Letter No. MGM-ECRHS/2018/62 dated October 27, 2018.

### Funding

None.

### Competing interests

The authors declare that they have no competing interests.

## Figures and Tables

**Table 1:**
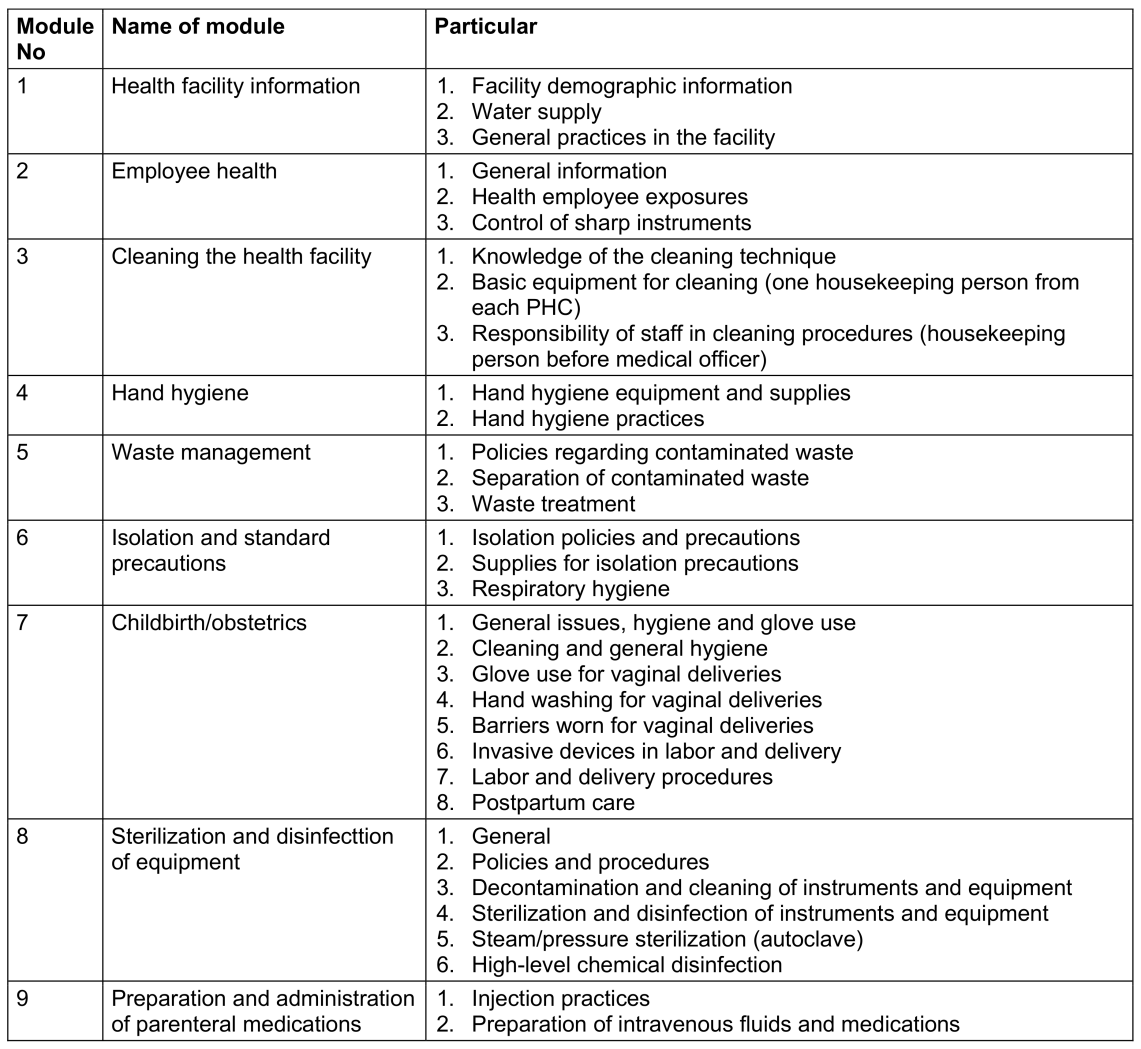
List of nine modules

**Table 2:**
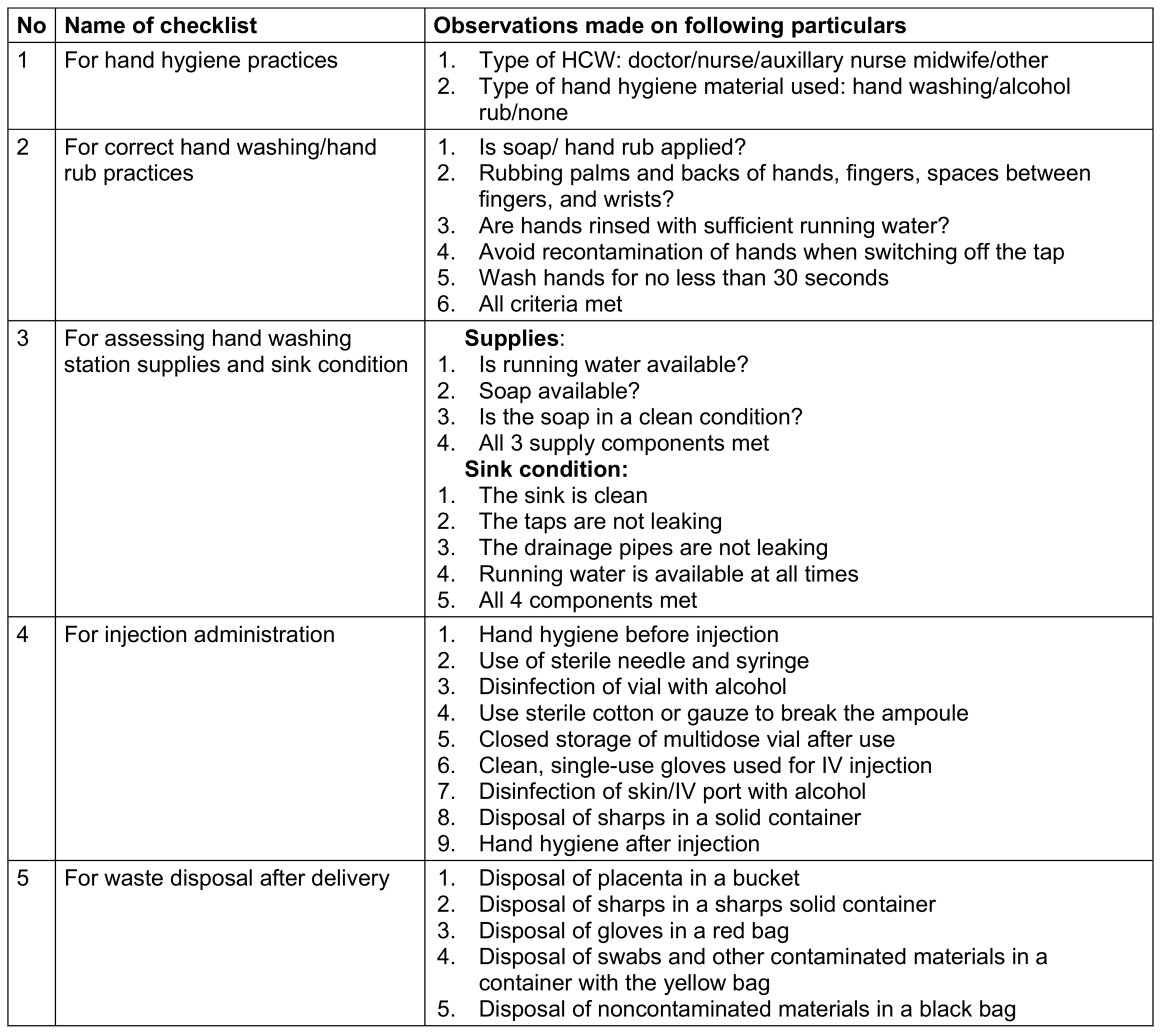
Checklist for observation of infection control practice

**Table 3:**
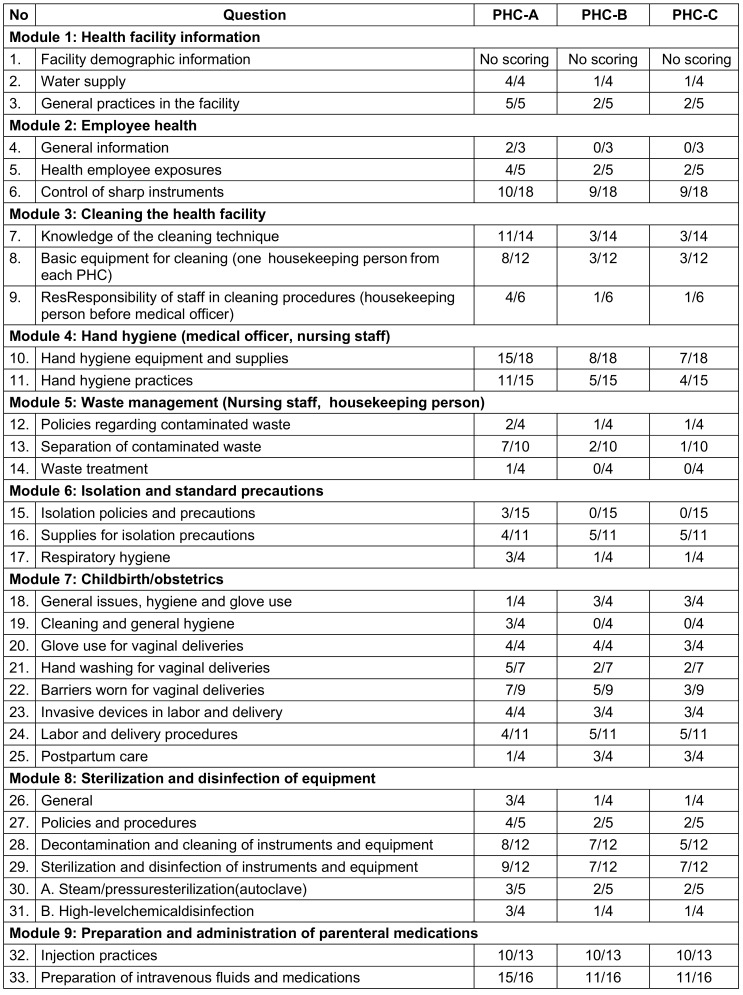
Scoring sheet of PHCs on the basis of nine modules

**Table 4:**
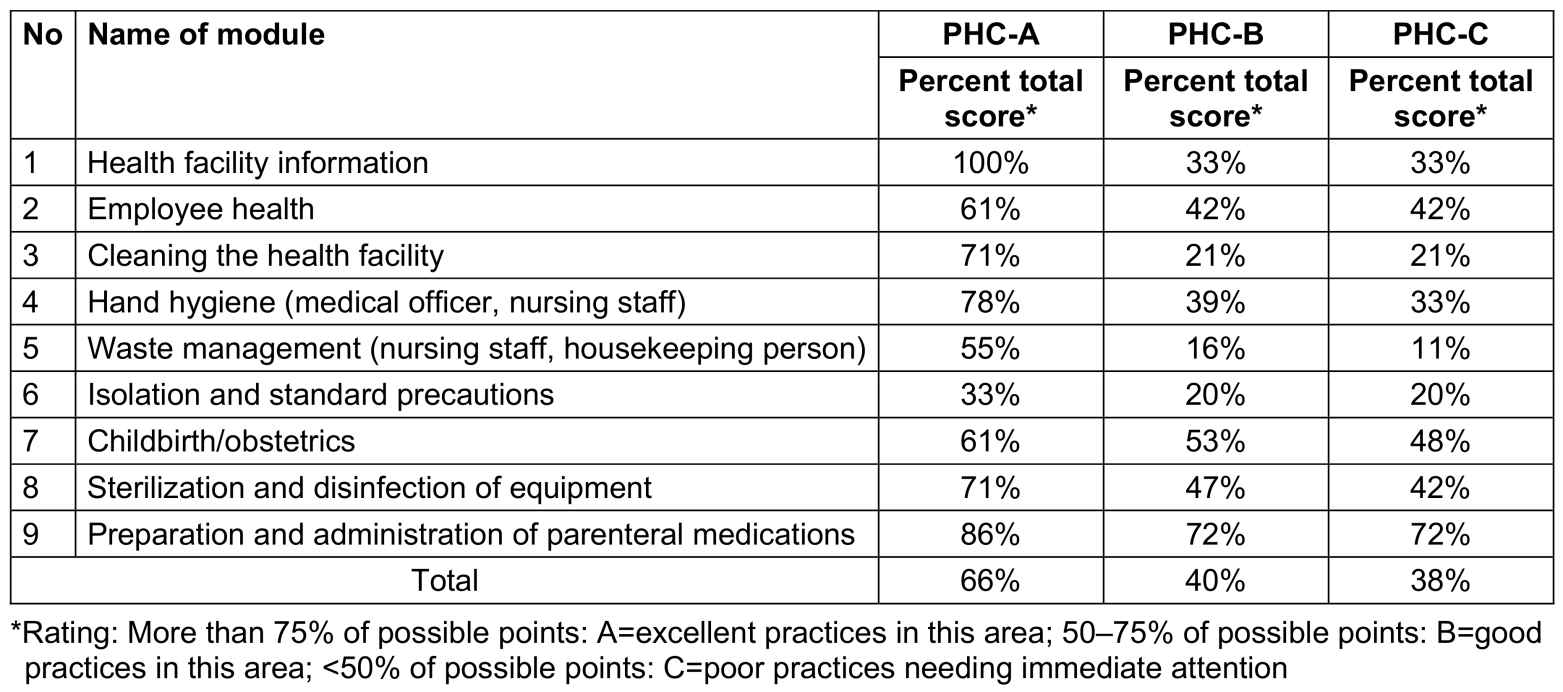
Assessment of scores* of all 3 PHC for 9 modules

**Table 5:**
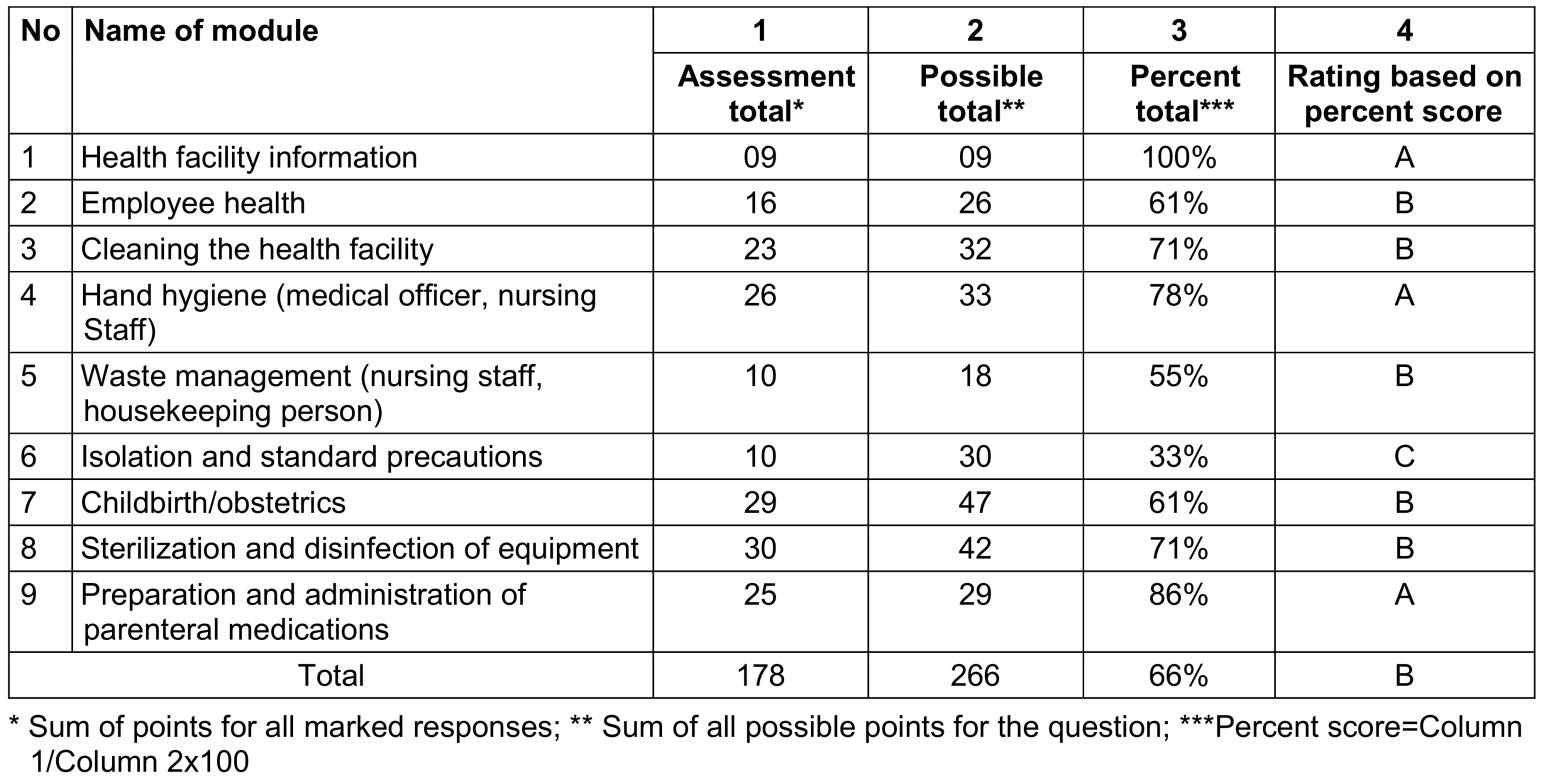
Assessment score of PHC-A (received a rating of A in 3 modules, B in 6 modules, and C in 1 module)

**Table 6:**
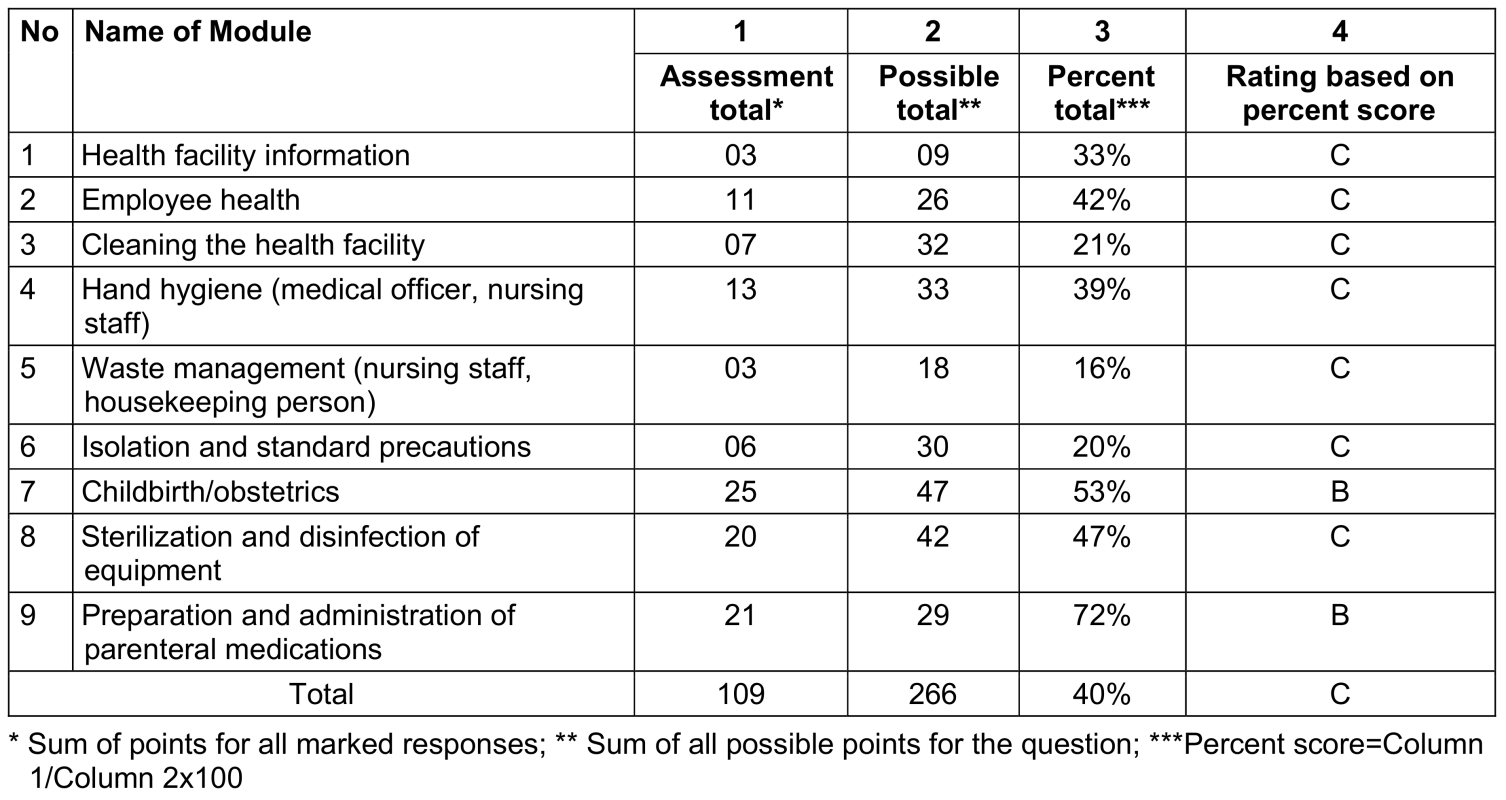
Assessment score of PHC-B (received a rating of B in the 2 modules and C in 7 modules)

**Table 7:**
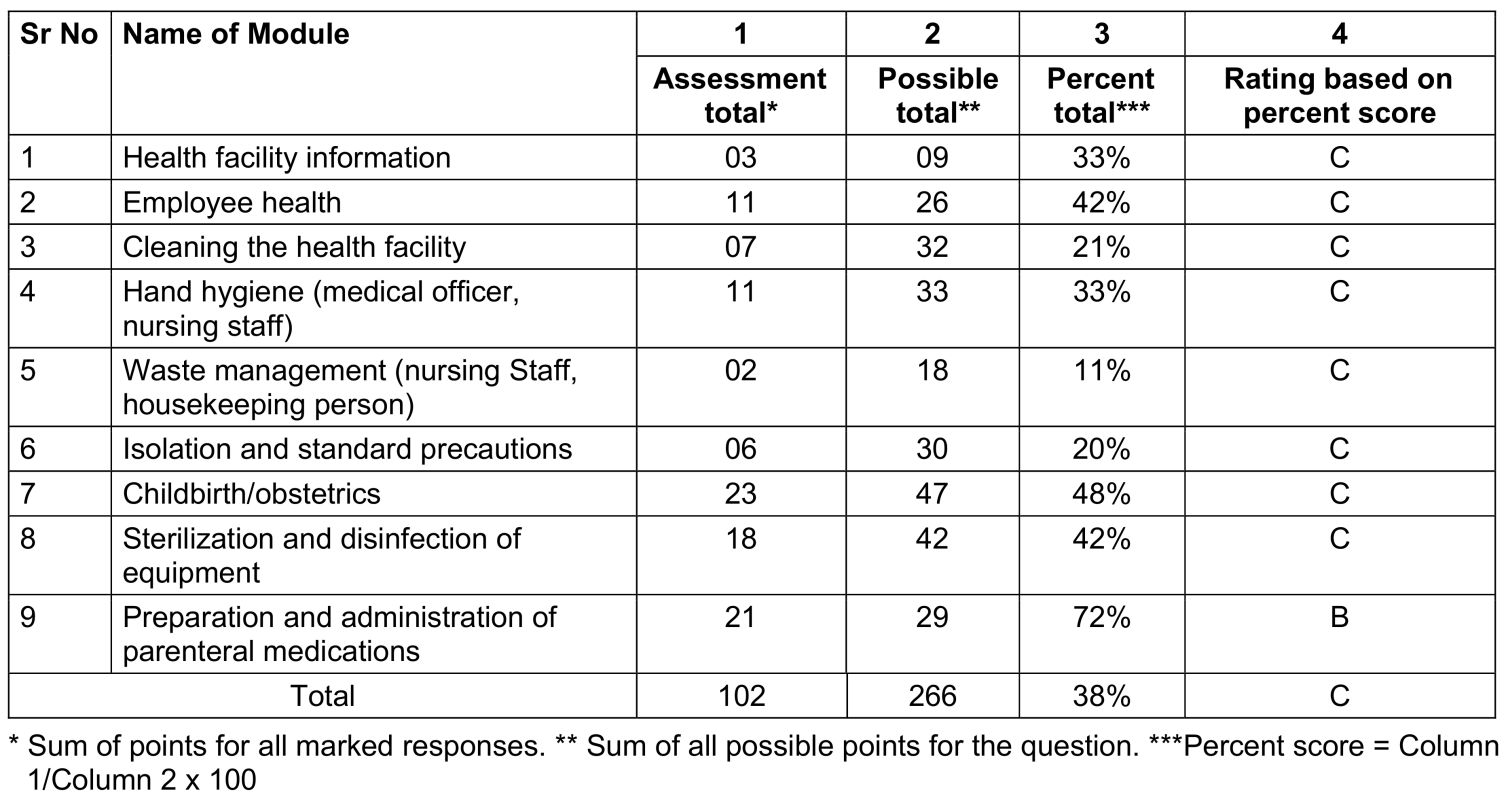
Assessment score of PHC-C (received a rating of B in 1 module and C in 8 modules)

**Table 8:**
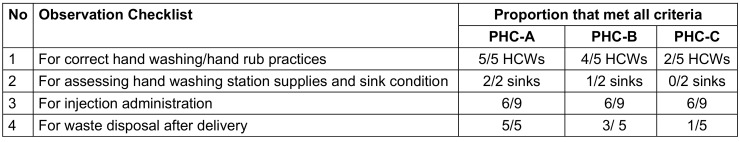
Analysis of observation checklist
